# Drivers-of-Liking (DOL) for Boiled Milk among Women of Reproductive Age and Children Aged between One and Five Years in Peri-Urban Communities in Ghana

**DOI:** 10.24966/FSN-1076/100034

**Published:** 2018-08-14

**Authors:** Maame Yaakwaah Blay Adjei, Joris Gerald Niilante Amissah, Angela Parry-Hanson Kunadu, Ezekiel Acquaah, Esi Colecraft, Gloria Ethel Otoo, Ernest Afrifa-Anane

**Affiliations:** 1 Department of Nutrition and Food Science, University of Ghana, Legon, Ghana; 2 Department of Family and Consumer Sciences, University of Ghana, Legon, Ghana; 3 Office of Research, Innovation and Development, University of Ghana, Legon, Ghana; 4 Regional Institute of Population Studies (RIPS), University of Ghana, Legon, Ghana

**Keywords:** Children, Consumption, Milk, Preference, Women

## Abstract

Raw milk is heat treated in different ways to improve the safety of the milk for consumption. The heat treatment imparts different sensory properties to the milk that may influence its acceptance by consumers. In Ghana, fresh milk is boiled and sold locally to consumers. Generally, consumption amongst women of reproductive age and children under five is low. In this study, the sensory properties that drive liking for local boiled milk and other heat-treated milks in women of reproductive age and children between 1 and 5 years of age was studied. External preference mapping was used to understand the sensory properties of the milks that were liked by the two consumer groups. The sensory properties of boiled milk that made women of reproductive age like a particular product was its sweet and salty taste, smooth mouthfeel and artificial flavour. For children between 1 and 5 years, the oily aftertaste, boiled egg aroma as well as its sweet taste made them like the same product as the adults. This product also had a cooked aroma and flavour which could have influenced liking in the consumer groups. A small minority of adult consumers liked products that had a smooth and runny appearance with a raw/uncooked note.

## Introduction

Ghana is a developing country facing malnutritional challenges amongst children and women of reproductive age [1-3]. The first thousand days of life can positively or negatively impact the long term health of an individual [4-6]. This means that the nutritional status of an adult woman is directly associated with nutritional intake at birth. It is therefore necessary to provide the needed nutrients for optimal growth of children at an early age so as to produce healthy adults.

Milk is an important food commodity when it comes to the provision of nutrients for growth and development. It contains high amounts of calcium for maximal bone mineralization; omega-3 fats which help prevent the onset of cardiovascular diseases; vitamins B, E, selenium and zinc which help in the regulation of blood sugar and the removal of free radicals from the body and also contain complete proteins to promote normal growth and development [7].

Despite the many health benefits of milk, milk consumption in developing countries like Ghana is relatively low compared with other developed countries. Although consumption of dairy products is on the rise in many urban communities in Ghana, generally, fresh milk consumption is very low. There are some communities in Ghana where diary activities may influence milk consumption patterns of consumers who live in these areas. Dairy communities in this context are defined as communities where dairying activities such as farming, processing and marketing occur. In Ghana, there are pockets of such areas typically in peri-urban communities.The demand for milk in these communities and most developing countries as a whole is expected to increase due to drivers like increasing income levels, urbanization, social and cultural factors.

Raw milk is often subjected to heat treatment to make it safe for consumption. The heat treatment imparts different sensory properties in the milk which may influence the acceptance of the milk for consumption. Cardello describes in vivid detail how this food-related phenomon leads to a measurable psychophysical behaviour [8]. In many dairying communities, raw milk is often boiled and sold locally as boiled milk. These heat-treated milks may have uncharacteristic sensory properties that make them different to industry processed heat-treated milks, which often have no off-flavour and are bland in flavour. Products such as the Ultra-High Temperature (UHT) treated milk, have characteristic cooked notes but are ‘clean’ with no other off flavours, they may have sweet or bitter taste [9]. In Ghana, these UHT milks are sold as fresh milk and are perceived to have improved hygiene due to its neat packaging. Local boiled milks are not packaged and are sold directly to consumers from the fire (with ice blocks to cool down the temperature) in milk markets or sold at farm gates as fresh pasteurised milk. There is thus a general perception that they are unsafe for consumption since milk is a fertile food for contamination with microorganisms [10]. Aside the safety issues, there may be some sensory barriers to the consumption of boiled milk amongst consumers in Ghana due to the uncharacteristic flavour notes that are typical in local boiled milks.

To promote the dairy market in Ghana, it is important to understande the sensory attributes that drive the acceptance and consumption of boiled milk amongst target consumers. A useful tool that can help to unearth the sensory properties of a product that drive liking in consumers is preference mapping.This method combines analytical data from descriptive work and consumer work to help understand what attributes in food are driving consumers to like or dislike food [11-13]. There are two basic approaches to preference mapping: Internal and external preference mapping methods. Both approaches have specific applications. Internal preference mapping focuses on the preference of consumers whiles external preference mapping focuses on understanding sensory information about the product and overlaying this information with consumer liking data [11]. In this study our main objective was to use external preference mapping tool to understand the sensory drivers for consumption of boiled milk in children under 5 years old and women of reproductive age (15-49 years). These consumer target groups are identified as nutritionally vulnerable groups in our society. Enhanced milk consumption amongst this target group would be ideal as the one single source food can provide the needed nutrients in two nutritionally vulnerable groups in society.

## Materials and Methods

### Samples and sample preparation

A total of eight boiled milk samples were evaluated. Six boiled milk samples were obtained from milk sellers and farm gates within the Greater Accra and Eastern Regions of Ghana. The milks obtained from milk markets were typically still simmering on fire (the milk is boiled in large aluminium pots on direct firewood). The temperature of the milk was always above 60°C in this state. Milk from the farm gate was often pasteurised by the farmer (pasteurisation conditions are not known). They were received chilled at <10°C. Commercially available Ultra-High Temperature (UHT) treated skimmed and full cream milks were obtained from local supermarkets and included in the product set to give a range of fluid milks available on the Ghanaian market. Boiled milk samples were received a day prior to the test day in pre-labelled sterilized glass bottles. They were stored in a refrigerator at 3-5°C throughout the test and were kept for no longer than 4 days after purchase. Microbiological safety tests confirmed that samples were still safe for consumption when kept under these conditions for 4 days. Assessors were served 20ml of each sample in 25ml transparent disposable cups at (20±2)°C. Trained assessors evaluated all 8 milk samples while consumers evaluated 6 out of the 8 samples.

### Test procedures

#### Consumer test

A total of 120 adult women of reproductive age between 15-49 years and 72 children less than age five were used for the study. Adult women and children were sampled from selected communities within the Greater Accra and Eastern Regions of Ghana, where fresh milk was typical available either due to the presence of dairying households or milk markets. The traditional 9-point hedonic scale with words and numbers was used by the adults whiles a 3-point category liking scale was used by the children (1=dislike very much, 2=neither like nor dislike and 3=like very much).

#### Descriptive test

A nine-member trained descriptive panel evaluated the boiled milk samples at the Sensory Evaluation Laboratory, University of Ghana using Sensory Spectrum (SS®) method. The panellists were selected based on time availability, good health conditions, and having no allergies to boiled milk. Panellists were trained for six weeks on descriptive test method using spectrum analysis method and on milk sensory properties before the final evaluation was done. Assessors were trained to describe, assess and score the intensities of the appearance, aroma, flavour, mouth feel and aftereffect of boiled milk samples. Assessors developed an agreed list of attributes that described the boiled milk samples using a consensus approach. Assessors completed individual scoring of intensities for the different attributes on 15cm intensity line scales using Compusense Cloud® (Compusensecloud (R), Guelph, Ontario, Canada). Samples were evaluated in triplicate.

### Statistical data analysis

All statistical analyses were done using XL-STAT (Addinsoft, France). Two-way (assessor X product) Analysis of Variance (ANOVA) was carried out on the sensory descriptive data to understand product differences of the 8 products selected. Consumer data was also analysed using two-way (assessor X product) Analysis of Variance (ANOVA) to determine differences in product liking scores. Where statistical significant differences were observed, LSD post-hoc analysis (Tukey’s HSD) was done to show where differences between products existed. Sensory product map based on descriptive data was generated using Principal Components Analysis (PCA) to show how products are distributed in the product space. The direction of consumer liking in the product space was determined based on consumer product map using PCA mapping technique and after cluster analysis on the consumer data. Agglomerative Hierarchical Cluster (AHC) analysis was used to group consumers into homogenous groups based on their liking score patterns.

To determine the drivers of liking for boiled milk, the external preference mapping tool in XL-STAT, (PREFMAP®) was used. In this method, the product map generated from the descriptive test is overlaid with consumer preference data from the acceptance test. In this study, the consumer cluster groups were used in the analysis and only the same six samples tested by consumers was used to generate the product map for PREFMAP®.

## Results and Discussion

### Sensory profile of boiled milk and product map

A total of 35 sensory attributes were used to characterise the sensory profile of the 8 different milks based on the appearance (7 attributes), aroma (7 attributes), flavour (12 attributes), mouthfeel (2 attributes) and aftereffects (7 attributes). There were statistical significant differences between the samples for all attributes evaluated with the exception of mouthfeel descriptors, smooth and runny. All other sensory descriptors generated were significantly discrim-inating between the boiled milk samples (Table 1 and 2). Table 3 shows the sensory descriptive attributes for the products and the food reference material that relate to those attributes on a 15cm intensity line scale.

**Table 1:** Mean scores of boiled milk products based on sensory descriptors; (modalities-appearance, mouthfeel and aftertaste).

	**Products**							
**Sensory Descriptors**	**A**	**B**	**CD**	**E**	**F**	**G**	**H**
**Appearance**								
Smooth	148.34^a^	148.53^a^	145.13^a^	146.08^a^	146.67^a^	149.60^a^	148.70^a^	133.59^b^
Runny	142.33^a^	142.09^ab^	141.66^ab^	142.30^ab^	142.19^ab^	141.32^ab^	142.28^ab^	141.30^b^
Glossy	125.40^bcd^	129.03^abc^	120.04^d^	131.09^ab^	121.90^cd^	137.50^a^	129.40^abc^	117.75^d^
Opaque	148.04^bc^	148.42^b^	148.39	147.08^d^	148.39^b^	149.53^a^	148.07^bc^	147.45^a^
Cream	48.10^bc^	46.44^c^	60.44^bc^	52.47^bc^	47.86^bc^	61.66^b^	99.02^a^	53.9^6bc^
Fat droplets	24.92^b^	14.25^c^	21.36^bc^	15.49^c^	20.86^bc^	2.33^d^	2.02^d^	34.79^a^
Particles at the bottom	10.57^ab^	6.71^bc^	11.44^a^	1.78^d^	3.77^cd^	0.23^d^	0.20^d^	0.99^d^
**Mouthfeel**								
Smooth	148.27	142.61	145.19	148.10	148.05	144.23	148.56	148.35
Runny	142.29	142.17	141.84	142.25	142.36	142.09	141.95	142.18
**Aftertaste**								
Sweet	6.71^b^	2.32^b^	6.99^b^	5.91^b^	4.03^b^	8.00^b^	3.09^b^	46.09^a^
Salty	5.26^a^	1.28^b^	2.05^b^	2.71^b^	1.74^b^	1.69^b^	2.10^b^	2.57^b^
Salivation	22.68^a^	25.0^a^	24.53^a^	28.7l^a^	20.39^a^	27.48^a^	19.73^a^	24.74^a^
Oily	15.67^b^	10.19^bcd^	11.54^bc^	13.05^bc^	14.38^b^	6.91^cd^	4.33^d^	28.32^a^
Milky	23 27^cd^	12.28^e^	29.39^bc^	25.96^a^	24.26^a^	46.91^a^	17.24^de^	33.64^b^
Astringent	18.96^ab^	15.65^b^	20.68^ab^	16.87^ab^	25.31^a^	21.09^ab^	17.90^ab^	22.99^ab^
Fresh cow meat	16.43^b^	31.65^a^	17.10^b^	31.46^c^	33.77^a^	3.07^c^	10.40^bc^	4.81^c^

**Table 2:** Mean scores of boiled milk products based on sensory descriptors (modalities-aroma and flavour).

	Products							
**Sensory Descriptors**	A	B	C	D	E	F	G	H
**Aroma**								
Milky	73.55^c^	75.68^c^	75.16^c^	64.45^cd^	60.42^d^	138.64^a^	128.13^a^	102.23^b^
Cooked note	58.54^c^	56.78^c^	57.44^c^	53.84^c^	55.68^c^	93.32^a^	80.34^b^	79.62^b^
Artificial flavour	7.50^bc^	0.28^c^	0.44^c^	0.34^c^	0.43^c^	8.91^b^	2.13^bc^	75.46^a^
Cereal	5.84^bc^	23.12^a^	10.28^b^	9.56^b^	9.50^b^	7.33^b^	0.28^c^	6.32^bc^
Cowy/Meaty	28.65^cd^	45.64^b^	29.78^c^	59.90^a^	57.76^ab^	5.06^e^	17.19^de^	8.11^e^
Raw/Uncooked	22.62^cd^	67.20^a^	15.78^de^	42.72^b^	41.40^b^	7.32^e^	32.73^bc^	9.68^de^
Smoky	6.97^cd^	18.03^b^	63.41^a^	5.85^cd^	16.70^bc^	2.43^d^	0.35^d^	7.73^bcd^
**Flavour**								
Sweet	14.47^b^	9.66^b^	12.64^b^	13.77^b^	9.18^b^	12.16^b^	9.09^b^	87.88^a^
Salty	8.35^a^	4.99^b^	5.30^b^	5.04^b^	3.65^b^	4.36^b^	3.55^b^	5.93^ab^
Milky 1	36.99^b^	10.12^cd^	20.72^c^	9.38^cd^	12.52^cd^	9.39^cd^	6.09^d^	78.55^a^
Milky 2	63.59^b^	65.09^b^	65.08^b^	86.72^a^	88.81^a^	4.73^c^	6.29^c^	9.64^c^
Milky 3	14.84^e^	31.74^cd^	38.50^c^	16.50^de^	16.23^de^	127.67^a^	104.08^b^	23.31^cde^
Cowy/Meaty	40.48^b^	56.53^a^	38.74^b^	59.17^a^	66.46^a^	4.13^d^	16.89^c^	8.79^cd^
Raw cow meat	30.91^c^	51.09^b^	31.36^c^	48.68^b^	60.96^a^	6.33^d^	22.12^c^	5.09^d^
Artificial flavour	5.89^b^	0.37^b^	0.36^b^	0.38^b^	0.20^b^	4.63^b^	0.41^b^	79.58^a^
Eggy 1	5.02^ab^	7.09^ab^	7.38^ab^	10.30^a^	9.90^a^	1.94^b^	6.49^ab^	11.20^a^
Eggy 2	10.98^bc^	2.15^c^	2.11^c^	2.22^c^	6.59^bc^	17.32^b^	35.09^a^	11.39^bc^
Cereal	12.17^b^	22.63^a^	9.44^bc^	10.48^b^	9.53^bc^	6.57^bc^	0.27^c^	8.54^bc^

Note: a-e Means within a row with the same superscript are not significantly different (p≤0.05).

**Table 3:** Sensory attributes of boiled milk products and their associated food reference materials on the intensity scale.

**Appearance**			
**Descriptor**	**Definition**	**Anchor**	**Scale/Reference**
Smooth	Absence of lumps in UHT milk	Not to very	7-lumps 10-curds 15-UHT milk
Cream colour	Characteristic cream colour associated with laughing cow cheese	Not to very	2-UHT milk 8-laughing cow cheese 15-ideal milk (evaporated)
Runny	Ability to flow easily like water	Not to very	1-ketchup 5-honey 15-water
Opaque	Inability to see through	Not to very	15-UHT milk
Glossy	Having a shiny surface associated with UHT milk	Dull to glossy	7-laughing cow cheese 15-UHT milk
Fatty droplets	Fat particles on the surface of milk	Not to very	0-absent 5-present
Particles at the bottom of milk sample	Presence of black particles at the bottom of milk sample	Not to very	0-absent 2-present
**Aroma**			
**Descriptor**	**Definition**	**Anchor**	**Scale/Reference**
Milky	Characteristic aroma of milk	Not to very	5-fresh milk 10-powdered milk 15-full cream milk
Meaty/Cowy	Characteristic aroma of fresh cow meat	Not to very	
Cooked note	Aroma of milk	Not to very	5-UHT milk 10-UHT milk cooked for 90 seconds 15-cooked evaporated milk
Artificial flavour/Essence	Aroma of vanilla and caramel sweet essence	Not to very	
Raw/Uncooked	Pungent aroma associated with raw meat and raw egg	Not to very	5-partially scrambled 7-boiled egg 10-raw egg 13-scrambled egg
Smoky	Aroma of uncooked turkey bacon	Not to very	6-smoked mackerel 9-unccoked turkey bacon 13-lit wood chips steeped in full cream milk for 20 minutes
Cereal	Aroma associated with processed maize product	Not to very	
**Flavour**			
**Descriptor**	**Definition**	**Anchor**	**Scale/ Reference**
Sweet	Basic sweet taste	Not to very	
Meaty/Cowy	Flavour of fresh cow meat	Not to very	
Smoky	Aroma of uncooked turkey bacon	Not to very	6-smoked mackerel 13-lit wood chips steeped in full cream milk for 20 minutes
Salty	Basic salt taste	Not to very	
Milky 1 Milky 2 Milky 3	Flavour of powdered milk Flavour of fresh cow milk Flavour of full cream milk	Not to very	Milky 1: 10-powdered milk Milky 2: 11-fresh milk Milky 3: 14-full cream milk
Cooked note	Flavour of boiled milk	Not to very	5-UHT milk 10-UHT milk cooked for 90 seconds 15-cooked evaporated milk
Raw/Uncooked	Pungent flavour of raw meat, raw egg	Not to very	5-partially scrambled 7-boiled egg 10-raw egg 13-scrambled egg
Cereal	Reminiscent flavour of boiled maize	Not to very	
Eggy 1 Eggy 2	Reminiscent flavour of boiled egg Reminiscent taste of raw egg	Not to very	Eggy 1: 6-boiled egg 12-scrambled egg Eggy 2: 5-partially scrambled eggs 10-raw egg
**Mouthfeel**			
**Descriptor**	**Definition**		**Anchor**
Smooth	Absence of lumps	Not to very	
Runny	Ability to flow easily in the mouth	Not to very	
**Aftertaste**			
**Descriptor**	**Definition**	Not to very	
Salivation	Production of saliva in the mouth	Not to very	
Fresh cow meat	Lingering cowy note in the mouth	Not to very	
Oily	Oily coating in the mouth	Not to very	
Sweet	Basic sweet taste	Not to very	
Milky	Lingering milk taste in the mouth	Not to very	
Astringent	Dryness in the mouth	Not to very	
Salty	Basic salt taste	Not to very	

**Figure 1:**
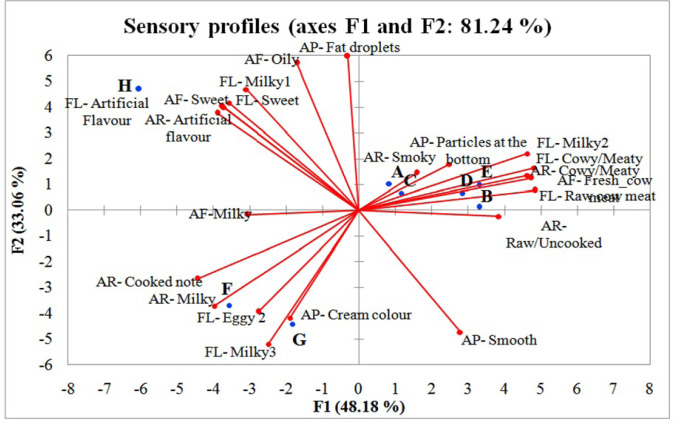
Sensory product map for boiled milk purchased from different processors.

The sensory product map for the 8 milk samples is shown in figure 1. The PCA product map shows that 81% of total variance in the data was explained in the first two dimensions as such meaningful interpretations can be made about the product positions in this dimension. Factor 1 (48%) is driven by the cooked aroma in the negative direction and raw/uncooked aroma in the positive direction while Factor 2 (33%) is driven by smooth appearance in the negative direction and particulate in the positive direction. The products were loaded in three distinct areas on the product map with specific attributes characterising each of these areas. Product H was the only product loaded in the space characterised by milky 1 (powdered milk flavour). Other attributes that define this space include sweet taste and after taste, artificial flavour note aroma and flavour, and fat droplets in appearance and oily aftertaste. Products F and G loaded in the product space characterised as milky 3 (full cream milk flavour). In this space, product F was associated more with milky aroma while product G was associated more with the milky 3 flavour (full cream milk). Other attributes characteristic of this space include eggy 2 flavour (raw egg flavour) and cream colour in appearance. The remaining 5 products loaded in the product space characterised by milky 2 (fresh cow milk flavour). Other characteristic attributes of products in this space were raw/uncooked aroma, rawcowy/meat aroma and flavour and fresh cow meat aroma. Smoky aroma is poorly loaded in either dimension 1 or 2 but has a stronger association with dimension 3 which is not shown here. Products A and C were associated more with smoky aroma while products B, D and E were associated more with the raw/ uncooked aroma and flavour notes.

**Key:** Letters A-H represent the 8 different products used. AP-Appearance, AF-Aftertaste, FL-Flavour, AR-Aroma

### Consumer acceptance of boiled milk products

**Figure 2:**
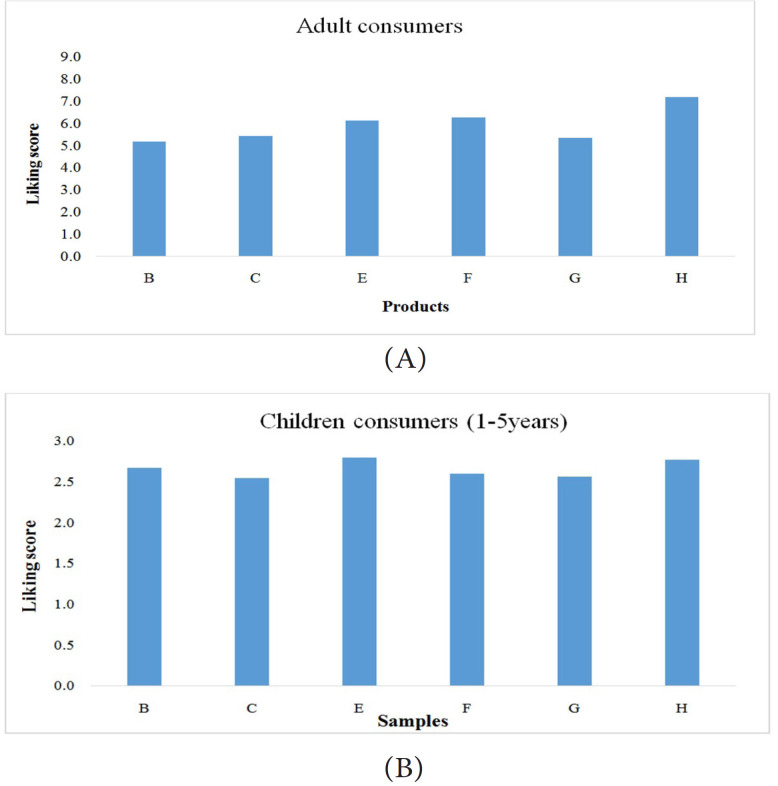
(A) Mean overall liking pattern of 120 adult consumers for boiled milk samples (1=dislike extremely to 9=like extremely); (B) Mean overall liking pattern of 72 children consumers (1-5 years) for boiled milk samples (1=dislike very much, 2=neither like nor dislike and 3=like very much).

**Figure 3:**
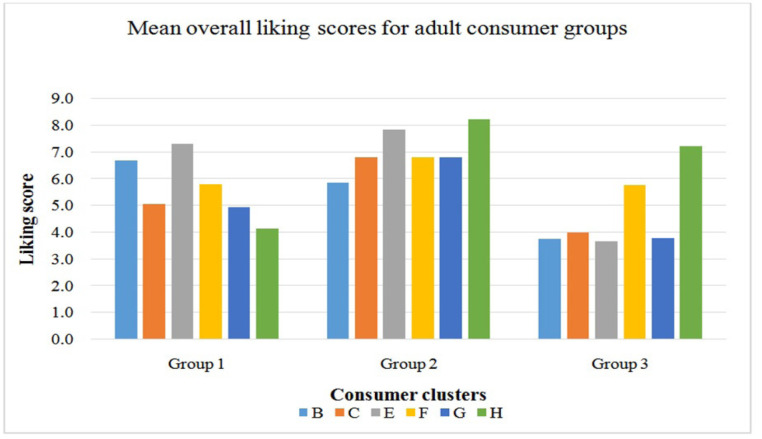
Mean overall liking scores of adult consumer groups after AHC was done (1=dislike extremely to 9=like extremely).

**Figure 4:**
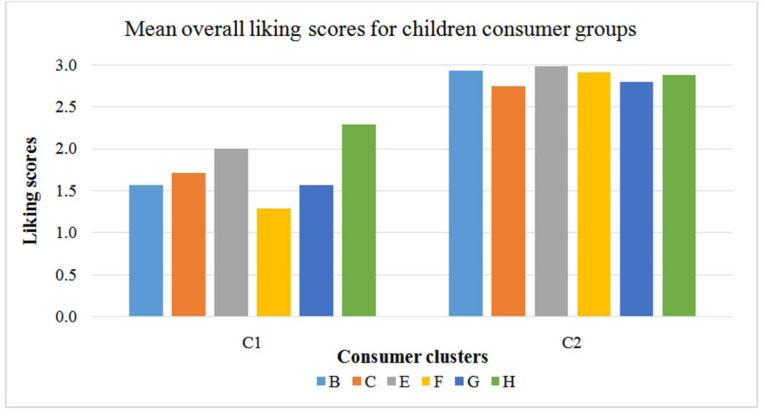
Mean overall liking scores of children consumer groups after AHC (1=dislike very much, 2= neither like nor dislike and 3=like very much).

Adult and child consumers evaluated 6 products out of the 8 milk products (B, C, E, F, G and H) as they were a good representation of the product space for boiled milks in Ghana without overloading their senses due to sensory fatigue. Adult consumers used the traditional 9-point hedonic scale while the children used a 3-point liking scale for the evaluations. Mean liking scores for the six products tasted by both adults and children are presented in the figures 2 ,3 and 4.

Although the overall liking pattern for adults and children differed, both groups liked product H more than the other products in the set. While the adults generally did not like product B, the children liked this product almost as much as product H. The least preferred products for the children were for products C and G. These products were also not liked much by the adult consumers.

Agglomerative Hierarchical Cluster (AHC) analysis identified three consumer cluster groups for the adult consumers based on their overall liking scores. Group 1 had 16% of consumers, group 2 had 45% of consumers while group 3 had 39% consumers. Groups 2 and 3 consumers both liked product H, however group 3 consumers were generally low product likers while group 2 consumers were high product likers. Group 1 consumers did not like product H overall.

The children consumers were placed into two clusters after AHC. Group 1 (C1) had the largest percentage of consumer, 81% and group 2 (C2) had only 19% of consumers. Similar to the adult consumers, both groups liked product H the most. However, group C2 consumers were non-discriminating in their liking for the products and scored all 6 products highly. The product liking pattern for group C1 consumers was similar to the adult consumers in group 2 who were also the majority of the adult consumers (45%). However, the children liked the products comparatively less than the adults.

## Drivers of liking

**Figure 5:**
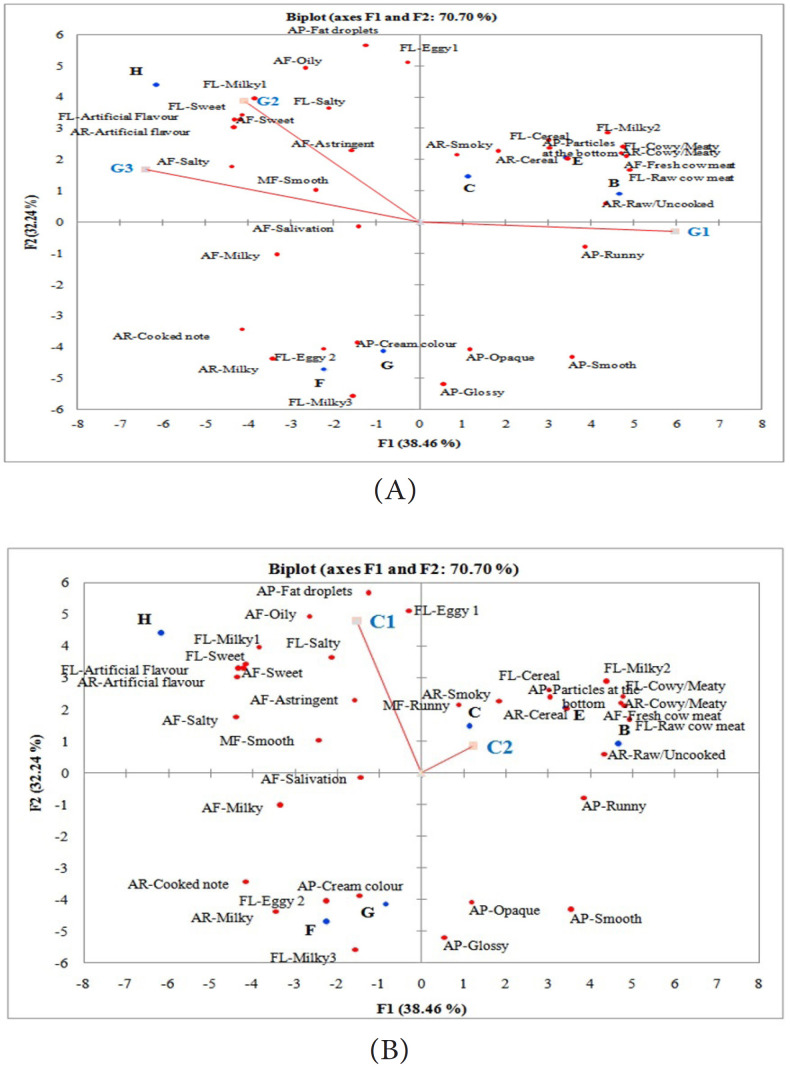
Preference mapping-Principal component biplot showing consumer perception of boiled milk. (A) Overall mean liking scores of the 3 adult consumer clusters. Vectors for the liking scores for the clusters as well as points for the 6 boiled milk samples are shown. G1, G2 & G3=adult consumer cluster 1, 2 and 3 respectively); (B) Vectors of the overall mean liking scores for the children clusters. C1=cluster 1, the largest cluster of the consumer data (81%) and C2=cluster 2 (19%).

The mean liking scores for the consumer groups were regressed onto the factors in the sensory product map in an external preference mapping analysis to determine drivers of liking for both the adult and children consumer groups. From the biplot in figure 5A, it is clear that the majority of adult consumers (groups G2 and G3), preferred product H which was sweet and salty in taste and aftertaste, had an artificial aroma and flavour, milky 1 (powdered milk) flavour and smooth mouthfeel. Although both group G2 and G3 liked similar products, there were slight differences in the attributes that drive liking for these groups. In G2 consumers, sweet taste and aftertaste were important attributes that drive liking while for G3 consumers, salty taste and smooth mouthfeel were important to drive liking. For group G1 adult consumers, there was no clearly liked product in the direction of their product liking based on the preference map (Figure 5B). What is clear though is that, they distinctly disliked product H as the direction of product liking for this group was opposite to that of groups G2 and G3. Group G1 consumers would like a product what is smooth and runny in appearance. They also appreciate products that have the raw/ uncooked aroma and flavour notes as was found in product B and E.

Preference mapping analysis of the children consumer groups showed that the direction of liking for majority of the children group C1, was in the direction of product H. However, the attributes of product H that drive liking for the children was not the same as for the adults. Contrarily in the children, sweet taste and aftertaste were not as strongly associated with their direction of liking as it was with the adults. It is possible that the intensity of sweetness was not high enough for the children’s palate compared with adults. This is not surprising as it was well known in the literature that adults and children have different levels of liking for sweetness intensity [14-16]. For the children in C2, it appears that the oily aftertaste, oil droplets and eggy 1 (boiled egg) flavour of the milk were important attributes that influenced their liking for product H. These attributes together may give a sensation of fullness in the mouth when consumed. The direction of liking for children group C2 was however not well explained in this product map, considering that the liking preference for consumers in this group was not well differentiated by these consumers.

A significant finding was that neither the adult or children consumer groups had the direction of product liking loading towards products F and G. This is interesting as these two products were the ultra-high temperature treated milks that are sold on the market. These milks are perceived to be more safe for consumption, however their sensory attributes did not drive liking amongst either of the consumer groups tested in this study.

## Conclusion

Fresh milk consumption in Ghana is generally very low and particularly so amongst children and women of reproductive age. This study has provided insight into the sensory drivers that influence consumption of different types of boiled milk products on the local Ghanaian market. Significantly, attributes associated with products that were UHT treated (products F and G) in this study, did not drive liking amongst either consumer groups. Both adult women of reproductive age and children under five years liked the sample H although the attributes that drive liking for the two groups were different. For women of reproductive age, sweet and salty taste, artificial aroma and flavour and smooth mouth feel are important attributes for product liking while for children under five years, oily aftertaste, eggy 1 (boiled egg flavour) and oily droplets in appearance were important attributes that drive liking. For a small majority of adult women of reproductive age however, products that have a raw/uncooked aroma and smooth runny appearance were more desirable. A small majority of children consumers however were non-discriminating between the products and liked all the products about the same. The results of this study show that there is potential to develop the local fresh milk market in the Ghana as they have sensory properties that appeal to the target consumers used in this groups who are also a nutritionally vulnerable group in the population.
